# Metabolite Differences of Polyphenols in Different Litchi Cultivars (*Litchi chinensis* Sonn.) Based on Extensive Targeted Metabonomics

**DOI:** 10.3390/molecules26041181

**Published:** 2021-02-23

**Authors:** Nonghui Jiang, Huili Zhu, Wei Liu, Chao Fan, Feng Jin, Xu Xiang

**Affiliations:** Key Laboratory of South Subtropical Fruit Biology and Genetic Resource Utilization, Key Laboratory of Tropical and Subtropical Fruit Tree Researches, Institute of Fruit Tree Research, Guangdong Academy of Agricultural Sciences, Ministry of Agriculture, Guangzhou 510640, China; zhuhuili2021@163.com (H.Z.); liuwei1987ahnu@126.com (W.L.); gdfanchao@163.com (C.F.); jinfeng@ibcas.ac.cn (F.J.)

**Keywords:** widely targeted metabonomics, polyphenols, litchi, cultivars, UPLC-MS/MS

## Abstract

Litchi is an important fruit cultivated in tropical and subtropical areas with high nutritious and delicious flavor and the pulp is the main part of the fruit consumed. Previous studies found that litchi had high total phenol content and antioxidant activity, but most of them focused on the identification of single or a few phenolic components with a low throughput test, and the metabolic differences of cultivars are still unknown to a some extent. In this study we used widely targeted metabolome based on ultra-performance liquid chromatography coupled with mass spectrometry (UPLC-MS/MS) to analyze the polyphenol metabolites of five different genotypes of mature litchi fruit. A total of 126 polyphenol metabolites in eight categories were identified to reveal the composition and differences of polyphenol; 15 common differential metabolites and 20 specific differential metabolites to each cultivar were found for the first time. The results infer that flavonoids, flavonols, hydroxycinnamoyls and catechins are the main polyphenol metabolites of litchi pulp. Cluster analysis showed that there were three groups of polyphenols from high to low; early maturing Feizhixiao is a kind of high polyphenol content cultivars, especially in catechins, anthocyanins, flavonols, quinic acids and hydroxycinnamoyls. The polyphenols in the flesh of mature litchi are rich, and there are significant differences among cultivars; there was a level of correlation between the contents of phenolics and the maturity of litchi cultivars; the content of phenolics in early maturing litchi cultivars appeared higher than those of mid- to late-maturing cultivars. This experiment will provide significant reference information for cultivation, breeding, processing and consumption.

## 1. Introduction

Litchi (*Litchi chinensis* Sonn.) is a typical and subtropical evergreen fruit tree, its fruit is popular among consumers with its high nutrition and attractive flavor. It has been cultivated for more than 2000 years in China. The cultivated area and output both rank as the world’s largest. In 2018, the litchi cultivation area was 551.75 thousand hectares and its output reached 2.3 million tons in mainland China [1]. After long-term natural selection and artificial cultivation, the germplasm resources of litchi are extremely rich with a total number over 500, and more than 30 cultivars have been sold in the commercial market [2]. Identification and evaluation of germplasm resources in litchi is the premise of effective use of resources.

Polyphenols are one of the most abundant and widely distributed secondary metabolites in plants. They are usually classified into five categories: phenolic acids, flavonoids, stilbenes, coumarins, and tannins. Phenolic acids include hydroxybenzoic acid and hydroxycinnamic acid. Flavonoids can be subdivided into flavonols, flavonoids, flavanols, flavanones, anthocyanins, and isoflavones. Tannins contain hydrolyzed tannins and condensed tannins (proanthocyanidins) [3]. Studies have shown that litchi contains a large number of polyphenols [4,5] with antioxidant [6,7], anticancer [8,9], and hypoglycemic activities [10,11], and the ability to lower blood lipids [12] and enhance immunity. Taken together, these traits have important contributions to human health. With the vigorous development of Chinese medicine in recent years, an increasing number of scholars are focusing on the polyphenols in litchi.

As a supplement to transcriptomics and proteomics, metabonomics has been applied in many fields, such as agriculture [13], food science [14,15], and botany [16]. Widely targeted metabonomics is different from the existing metabolite detection methods and integrates the advantages of non-targeted and targeted metabolite detection technologies, such as high throughput, high sensitivity, high qualitative accuracy, good repeatability, and complete database availability. Metabonomics can play an important role in variety identification and selective breeding [13].

Although there are many previous studies on the evaluation of functional components of litchi, most of them have focused on the identification of single or a few phenolic components, and the throughput has been low. There is no report on the systematic analysis of metabolic characteristics and change rules from metabonomics. Understanding and exploring the differences of polyphenol metabolism among different genotypes of litchi is very important while the metabolic differences of different cultivars are still unknown to some extent. In view of the fact that the flesh of litchi is the largest edible part, we used ultra-performance liquid chromatography coupled with mass spectrometry (UPLC-MS/MS) to compare and analyze the polyphenol metabolites of five different genotypes of mature litchi fruit. We screened the different metabolites to provide a theoretical basis for cultivar identification, breeding, cultivation, and production and processing of litchi.

## 2. Results

### 2.1. Qualitative/Quantitative Analysis of Metabolites and Quality Control Analysis

**Figure 1:**
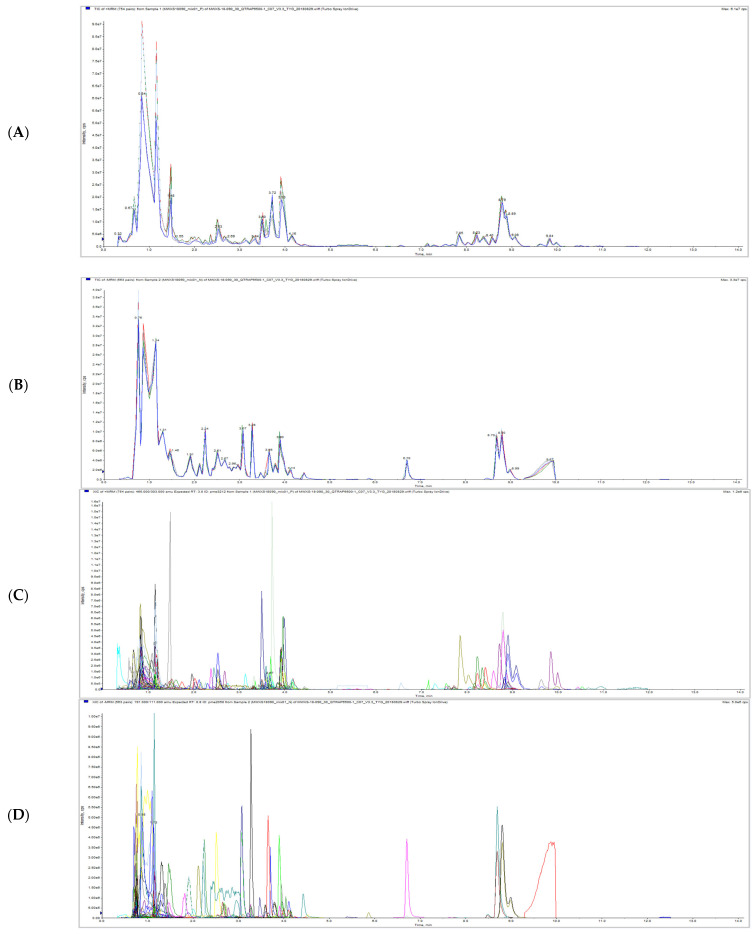
Total ions current overlap diagram (**A**,**B**) and multi-peak detection plot of metabolites in the multiple reaction monitoring mode (**C**,**D**) of litchi QC samples detected by mass spectrometry. Note: (**A**,**C**) is a positive ion mode, (**B**,**D**) is a negative ion mode and each mass spectrum peak with different colors represents a metabolite detected. The abscissa is the retention time (RT) of metabolite detection, and the ordinate is the ion current intensity (CPS, count per second) of ion detection.

The total ion current (TIC) overlap diagram (Figure 1A,B) and multi-peak detection plot of metabolites in the MRM (Figure 1C,D) from MS analysis of mixed quality control sample (QC) were analyzed by overlapping display analysis. The results showed that the total ion flow curve of metabolite detection had high overlap; that is, the retention time and peak intensity were consistent, indicating that the instrument signal stability was good.

Based on the MVDB database, the metabolites of the samples were analyzed qualitatively and quantitatively by MS. The multiple reaction monitoring mode (MRM) metabolite detection multi peak diagram showed the substances that can be detected in the sample. The peak area of each chromatographic peak represented the relative content of the corresponding substance. Finally, the integral data of all chromatographic peak areas were derived and saved, the metabolite number, integral value, and corresponding metabolite name of some metabolites detected in this experiment were shown in Table S1.

### 2.2. UPLC-MS/MS Analysis

**Figure 2:**
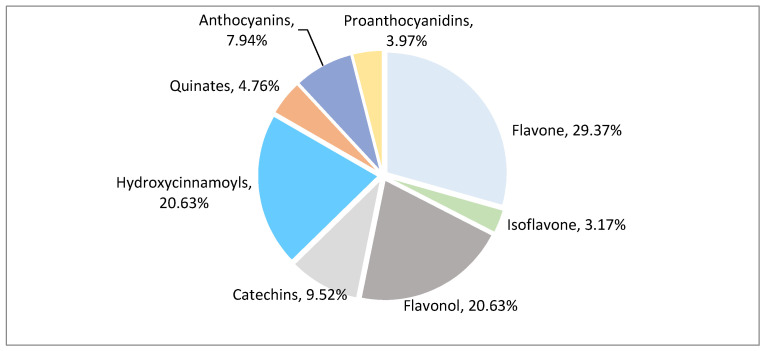
Classification pie chart of polyphenol metabolites in different litchi cultivars.

After qualitative and quantitative analysis by MS, a total of 126 polyphenol metabolites were detected from eight polyphenol categories (Figure 2, Table S1). There were 37 flavonoids (accounting for 29.37% of total polyphenol metabolites), 26 flavonols (20.63%), 26 hydroxycinnamoyls (20.63%), 12 catechins (9.52%), 10 anthocyanins (7.94%), 6 quinic acids (4.76%), 5 proanthocyanidins (3.97%), and 4 isoflavones (3.17%). Flavonoids, flavonols, hydroxycinnamoyls, and catechins accounted for 80.23% of the total polyphenols, indicating that these compounds are the main polyphenol metabolites in litchi.

**Figure 3:**
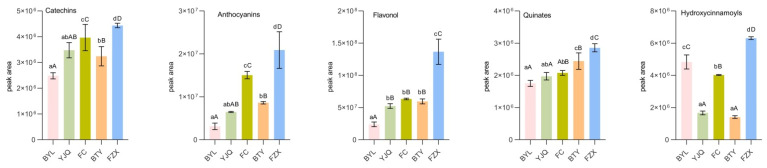

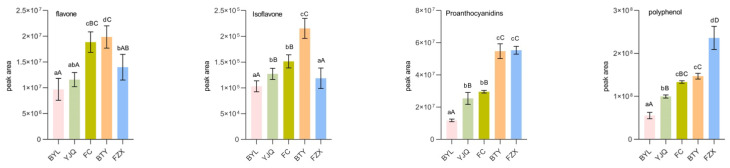
Comparison of polyphenol metabolites in different litchi cultivars. Note: The data of similar category of polyphenol metabolite obtained by summarizing the peak areas of each metabolites of the same kind, total phenols are the sum of phenolic compounds. Small letters means significant differences at the level of 0.05; capital letters means extremely significant differences at the level of 0.01, the same small letters or capital letters mean no significant differences at the level of 0.05 or 0.01.

Comparing the peak areas of catechins, anthocyanins, flavonols, quinines, hydroxycinnamoyls, proanthocyanidins, flavonoids, and isoflavones in different litchi cultivars (Figure 3), the catechins, anthocyanins, flavonols, quinics, hydroxycinnamoyls, and total polyphenols (sum of the above eight kinds phenolic compounds) in early maturing FZX were significantly higher than those in other cultivars (*p* < 0.01); its proanthocyanidins were significantly higher than that of Medium Maturing YJQ, FC, and late-maturing BYL; flavonoids and isoflavones in BTY were significantly higher than those in YJQ, FC, and BYL (*p* < 0.05). However, the contents of seven kinds polyphenols except hydroxycinnamoyls in late-maturing BYL were lower than those in other cultivars, especially in flavonols, proanthocyanidins, isoflavones and total polyphenols (*p* < 0.01). The results showed that there was a level of correlation between the content of polyphenols and the maturity of litchi cultivars. The contents at early ripening were higher than those at late ripening in general.

### 2.3. Multivariate Statistical Analysis of Metabonomics

#### 2.3.1. Principal Component Analysis (PCA)

**Figure 4:**
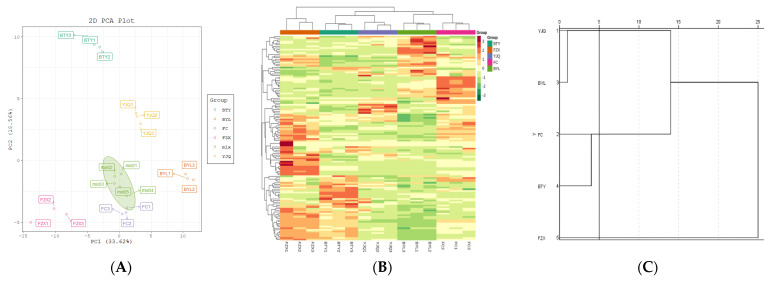
PCA score plot and cluster map of polyphenol metabolites in different litchi cultivars. (**A**) PCA score plot; (**B**) Cluster heat map. The color sequence from red to dark green indicates metabolites abundance from high to low; (**C**) Cluster analysis chart, genealogy using average join (between groups), rescaled distance clustering combination.

A total of 126 polyphenol metabolites of five different litchi cultivars were analyzed by PCA (PC1: 33.62%, PC2: 18.56%, PC3: 14.05%) (Figure 4A). The results showed that there were significant differences in the metabolites of different litchi cultivars. PCA could clearly separate the cultivars from the QC samples, indicating that the data processing for each sample was repeatable and reliable. Two early-maturing cultivars (FZX and BTY) were mainly distributed in the negative half axis of PC1, and the mid- to late-maturing cultivars (FC, YJQ, and BYL) were mainly distributed in the positive half axis of PC1. This showed that there were differences in metabolic profiles between early-maturing cultivars and mid- to late-maturing cultivars.

#### 2.3.2. Cluster Analysis

The R program was used to draw the cluster heat map which shows the hierarchical cluster analysis results of 126 polyphenol metabolites of 5 litchi cultivars; the metabolites with the same characteristics were classified by hierarchical clustering analysis, and the inter group variation of metabolites characteristics was identified (Figure 4B); the figure shows that there is an obvious grouping pattern among different cultivars; when Euclidean distance is 5, there are three groups among cultivars (Figure 4C): the first group is FZX with higher polyphenol content, the second group is BTY and FC with the middle content of polyphenols, and the third group is YJQ and BTY with lower content of polyphenols.

#### 2.3.3. Orthogonal Projections to Latent Structures-Discriminant Analysis (OPLS-DA)

Taking FZX as the reference cultivar, the samples of different cultivars were compared in four groups: FZX vs. BTY, FZX vs. YJQ, FZX vs. FC, and FZX vs. BYL. To maximize the differentiation among groups and screen the differential metabolites, the OPLS-DA model was established using multidimensional statistics (Figure S1). OPLS-DA shows that there is large variability among the different groups and among the samples within the group. The R2 and Q2 values of the OPLS-DA model are high in each group (Figure S2), which shows that the models have good prediction ability and reliability. The alignment verification of OPLS-DA (*n* = 200) shows that R2Y and Q2 are less than R2Y and Q2 of the original model, which indicates that the model is meaningful. Thus, it is feasible to group and compare the metabolites of different cultivars with reference to FZX, and there are obvious differences among the groups. Therefore, we can screen the different metabolites according to variable importance projection (VIP) value.

### 2.4. Screening and Identification of Different Metabolites

**Figure 5:**
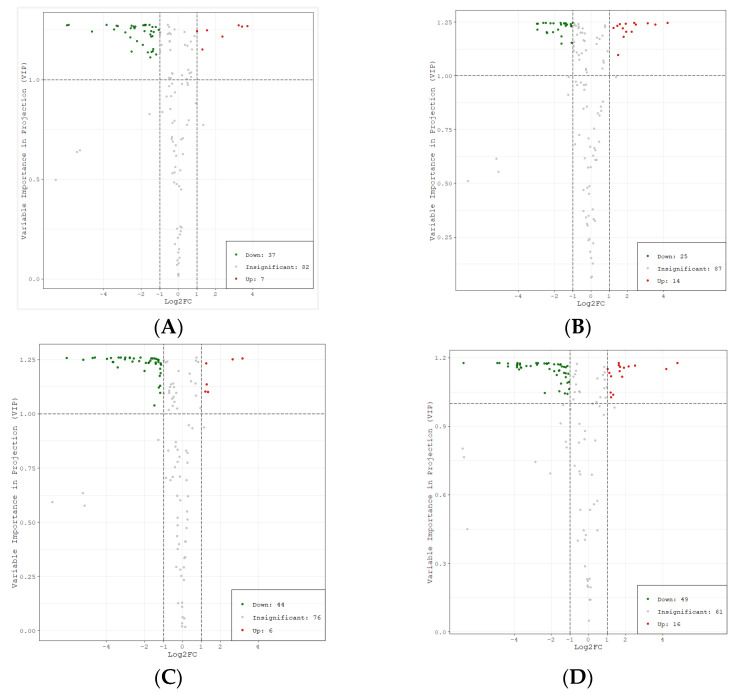
Volcano plots of different metabolites among litchi cultivars. Note: (**A**) BTY vs. FZX (**B**) FC vs. FZX (**C**) YJQ vs. FZX (**D**) BYL vs. FZX. Each point in the figure represents a metabolite; the abscissa represents the logarithm value of the multiple of quantitative difference of a metabolite in two samples, and the ordinate represents the VIP value. Fold change ≥2 indicates that the difference is up-regulated, and fold change ≤0.5 indicates that the difference is down-regulated. The green dot represents the down-regulated differential metabolite; the red dot represents the up-regulated differential metabolite, and the gray dots represent detected but insignificant metabolites.

Based on the results of OPLS-DA, the different metabolites among different cultivars were screened. At the same time, metabolites with significant difference were selected according to the *P* value of univariate analysis, fold change ≥ 2, fold change ≤ 0.5, and VIP ≥ 1. According to the VIP and fold change values, the volcano plots of different metabolites were drawn (Figure 5).

The statistics of significant difference for metabolites were shown in Table 1. The number of metabolites with a significant difference between groups showed the trend BYL vs. FZX > YJQ vs. FZX > BTY vs. FZX > FC vs. FZX, while the number of metabolites with no significant difference was the opposite, showing that the lower variability of FC and BTY compare with FZX, while the higher variabilities of BTY and YJQ compare with FZX. There was a level of correlation between the number of different metabolites in the groups and the maturity of the cultivars; that is, the closer to the maturity of FZX with early maturing, the smaller the difference. The number of down-regulated metabolites was greater than the number of up-regulated metabolites, indicating that there were more polyphenol metabolites with significant difference in FZX than in other cultivars.

**Figure 6:**
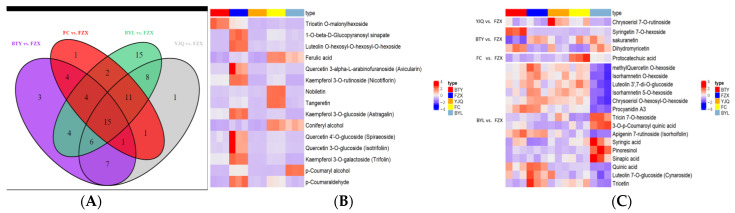
(**A**) Wayne diagram, (**B**) heat map of common differential metabolites, and (**C**) specific differential metabolites. The color sequence from red to dark blue indicates metabolites abundance from high to low in heat map.

According to the Wayne diagram (Figure 6A), there were 15 different polyphenol metabolites that were common among the different litchi cultivars. These compounds were tricetin *O*-malonylhexoside, 1-*O*-β-d-Glucopyranosyl sinapate, luteolin *O*-hexosyl-*O*-hexosyl-*O*-hexoside, ferulic acid, quercetin 3-α-l-arabinofuranoside, kaempferol 3-*O*-rutinoside, nobiletin, tangeretin, kaempferol 3-*O*-glucoside(astragalin), coniferyl alcohol, quercetin 4′-*O*-glucoside(spiraeoside), quercetin 3-*O*-glucoside(isotrifoliin), kaempferol 3-*O*-galactoside(trifolin), *p*-coumaryl alcohol, *p*-coumaraldehyde, including 6 flavonols, 5 hydroxycinnamoyls, and 4 flavonoids.

After analyzing the 15 common metabolites (Figure 6B, Table S2), it was found that the contents of 1-*O*-β-d-glucopyranosyl sinapate, luteolin *O*-hexosyl-*O*-hexosyl-*O*-hexoside, kaempferol 3-*O*-rutoside, kaempferol 3-*O*-glucoside, kaempferol 3-*O*-galactoside, quercetin 3-α-l-arabinofuranoside and p-coumaraldehyde were significantly higher in FZX than in other cultivars (*p* < 0.05). The contents of ferulic acid, nobiletin in FC were significantly higher than in other cultivars (*p* < 0.05), while the content of *p*-coumaryl alcohol in BYL was significantly higher than in other cultivars (*p* < 0.01). For BTY, the level of tricetin *O*-malonylhexoside was significantly higher than that in other cultivars (*p* < 0.01). The number of polyphenol metabolites was different, which indicated that there were differences in the expression of the metabolic spectrum among the different cultivars of litchi.

In addition, 20 specific differential metabolites (9 flavonoids, 4 flavonols, 3 hydroxycinnamoyls, 2 quinines, 1 proanthocyanidins, and 1 catechin) to each variety were found. BTY contained syringetin 7-*O*-hexoside, sakuranetin, and dihydromyricetin; FC contained protocatechuic acid; YJQ contained chrysoeriol 7-*O*-rutinoside; and BYL contained methyl quercetin *O*-hexoside, isorhamnetin *O*-hexoside, luteolin 3’,7-di-*O*-glucoside, isorhamnetin 5-*O*-hexoside, chrysoeriol *O*-hexosyl-*O*-hexoside, proanthocyanidin A3, tricin 7-*O*-hexoside, 3-*O*-*p*-coumaroyl quinic acid, apigenin 7-rutinoside (isorhoifolin), syringic acid, pinoresinol, sinapic acid, quinic acid, luteolin 7-*O*-glucoside (cynaroside), and tricetin (Figure 6C, Table S3). BYL had the largest number of specific metabolites, with 5 up-regulated and 10 down regulated, followed by BTY with 2 up-regulated and 1 up-regulated, FC and YJQ with 1 up-regulated each. Flavonoids, flavonols, and hydroxycinnamoyls accounted for 88.6% of all common and unique metabolites, so they were the main sources of litchi differential metabolites.

## 3. Discussion

### 3.1. Identification of Polyphenol Metabolites in Litchi

The content of polyphenols in litchi pulp was 69.74–186.22 mg·100 g^−1^, and this was the main active ingredient in litchi pulp [17]. Thus, it is of vital significance to identify the polyphenol components of litchi. After HPLC analysis and identification, Su et al. [4] found that the phenolic compounds in litchi pulp were mainly composed of nine compounds that included 3,4-dihydroxybenzoic acid, catechin, vanillic acid, caffeic acid, syringic acid, epicatechin, high catechol, ferulic acid, and rutin. Of these compounds, catechol, rutin, and epicatechin were present in the highest concentrations. Zhang et al. [6] analyzed the phenolic components in litchi pericarp and found that epicatechin, proanthocyanidin B2, epigallocatechin, and proanthocyanidin B4 were the main components. Lv et al. [10] isolated and purified proanthocyanidin B2, kaempferol 3-*O*-rutoside, isorhamnetin 3-*O*-rutoside, quercetin 3-*O*-rutinoside-(1→2)-*O*-rhamnoside, kaempferol 3-*O*-rutoside, isorhamnetin 3-*O*-rutinoside-(1→2)-*O*-rhamnoside, and other phenolic substances from the flesh extract of FZX. Hu et al. [18] identified the soluble free phenols and bound phenols in litchi pulp, and described seven phenolic compounds: kaempferol 3-*O*-rutinoside, quercetin 3-*O*-rutoside rhamnoside, kaempferol 3-*O*-rutinoside-rhamnose, narcissus, rutin, quercetin, and kaempferol. Among them, quercetin 3-*O*-rutinoside rhamnoside had the highest polyphenol content in litchi pulp. Zhong et al. [19] used LC–ESI-MS/MS to identify polyphenols in litchi pulp and found epicatechin, proanthocyanidin B2 and its isomer, and proanthocyanidin trimer. These previous studies mainly identified a single phenolic compound or a few at most. In the present study, a wide-ranging analysis of polyphenol metabolites using targeted metabonomics was conducted for five different genotypes of litchi, and a total of 126 polyphenol metabolites from eight categories were detected. Flavonoids (37 compounds, 29.37%), flavonols (26, 20.63%), hydroxycinnamoyls (26, 20.63%) and catechins (12, 9.52%), which accounted for 80.23% of polyphenols in litchi, were the main polyphenol metabolites.

In this experiment, we identified a total of 37 flavonoids, mainly in the form of chrysoeriol, wheat flavone, luteolin and apigenin, which combined with hexose, rutinose and other glycosides. chrysoeriol, wheat flavone, luteolin, and apigenin are natural flavonoids, which exist in many plants and have anti-inflammatory, antiallergic, antioxidant, antitumor, antibacterial activities, and other functions [20,21,22,23,24,25].

A total of 26 flavonols were identified. Flavonols are a class of flavonoid compounds with the basic skeleton of 3-hydroxyflavone. They exist in plant cell fluid in glycosidic form. The most common examples are kaempferol, quercetin, myricetin and isorhamnetin. Flavonols play many roles in plants, such as protecting the plant from damage caused by ultraviolet radiation, regulating growth hormone transport, and regulating the color of pericarp or flower [26]. In this experiment, kaempferol, quercetin and isorhamnetin were the main flavonols in litchi, and hexoside, rutoside, and rhamnoside were the main glycosides. Dihydromyricetin and dihydrokaempferol, as intermediates of phenylpropane metabolism in litchi, play an important role in the synthesis of anthocyanins and proanthocyanidins.

In addition to proanthocyanidin A3, proanthocyanidin A1, proanthocyanidin A2, proanthocyanidin B2, and proanthocyanidin B3, the catechins such as l-epicatechin, epicatechin-epiafzelechin, epicatechin gallate (ECG), epigallocatechin (EGC), epigallate catechin gallate (EGCG), catechin, protocatechuic acid *O*-glucoside, (+)-gallocatechin (GC), gallocatechin-catechin, catechin-catechin-catechin, protocatechuic aldehyde, and protocatechuic acid were also identified. Proanthocyanidins are formed by condensation of different amounts of catechin, epicatechin, or gallic acid through C4–C6 or C4–C8 bonds. The simplest proanthocyanidins are epicatechin, catechin, or dimer, trimer, and decamer formed by epicatechin and catechin [27]. Therefore, if catechins, which are the structural units of proanthocyanidins are added, the types of proanthocyanidins in litchi pulp are more diverse. In this experimentproanthocyanidin B2 and proanthocyanidin B3 were significantly more abundant than proanthocyanidins A3, proanthocyanidins A1, and Proanthocyanidins A2 (*p* < 0.05). Proanthocyanidins are one of the best natural antioxidants found so far because of their abundant hydroxyl groups and strong antioxidant capacity [28].

Fifteen common metabolites (six flavonols, five hydroxycinnamoyls, and four flavonoids) were identified, among which kaempferol 3-*O*-rutoside, kaempferol 3-*O*-glucoside, and kaempferol 3-*O*-galactoside were kaempferol compounds in the form of glycosides. These compounds have anticancer activity [29], can be used to treat diabetes and osteoporosis [30,31], and can provide protection to damaged cells [32]. Quercetin 4′-*O*-glucoside, quercetin 3-*O*-glucoside, and quercetin 3-α-l-arabinofuranoside are quercetin compounds in the form of glycosides; they not only have antioxidant and free radical scavenging effects but also have anti-inflammatory, antiviral, antitumor, hypoglycemic, and immunomodulatory effects [33]. Ferulic acid, *p*-coumarin, and coniferol are hydroxycinnamic acids; these compounds terminate the chain reactions of free radicals, promote the production of free radical scavenging enzymes, increase the activity of glutathione thiotransferase and quinone reductase, and inhibit the activity of tyrosinase, thereby providing a role in the regulation of human physiological functions [34,35,36,37,38].

### 3.2. Differences of Polyphenol Metabolites in Different Litchi Cultivars

FZX is an early-maturing litchi cultivar with high polyphenol content [17]. In the present study, catechins, anthocyanins, flavonols, quinic acids, hydroxycinnamoyls, proanthocyanidins, and total polyphenols were much higher in FZX than in other cultivars (*p* < 0.01). The contents of flavonoids and isoflavones in BTY were significantly higher than those in other cultivars (*p* < 0.05). However, the contents of seven types of polyphenols except hydroxycinnamoyl in late-maturing BYL were lower than those in other cultivars. Among the common metabolites of FZX, 1-*O*-β-d-glucopyranosyl, luteolin *O*-hexosyl-*O*-hexosyl-*O*-hexoside, kaempferol 3-*O*-rutin, kaempferol 3-*O*-glucoside, kaempferol 3-*O*-glucoside, kaempferol 3-*O*-galactoside, and *p*-coumarin were significantly higher than those of other cultivars (*p* < 0.05). The contents of ferulic acid, lysimachin, and tangutin in FC were significantly higher than those in other cultivars (*p* < 0.05), while *p*-hydroxyphenylethanol was significantly higher in BYL than in other cultivars (*p* < 0.01) and malonyl hexoside was significantly higher in BTY than in other cultivars (*p* < 0.01).

The number of polyphenol metabolites was different between cultivars, which indicated that there were differences in the expression of metabolic spectrum among the litchi cultivars. The polyphenol contents of early-maturing FZX and BTY were higher than those of mid- to late-maturing cultivars of FC, YJQ, and BYL. The results showed that there was a level of correlation between the phenolic contents in litchi and the cultivars with different maturity type. This may be due to the significant positive correlation between the expression level of L. *chinensis* gene (*LcFLS*), which is the key enzyme of flavonol synthesis and flavonol in different maturity cultivars. There was a downward trend in *LcFLS* expression from early-maturity to late-maturing cultivars. *LcFLS* had regular differences among different maturity cultivars [39].

## 4. Materials and Methods

### 4.1. Materials and Instruments

**Figure 7:**
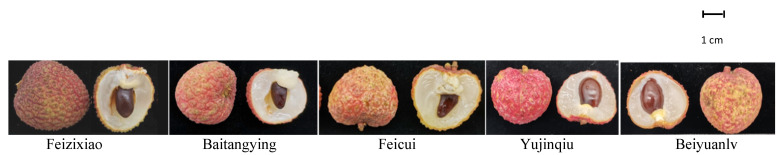
Image description of five Litchi cultivars.

From June to July 2018, five litchi cultivars (Figure 7) were selected from four cultivation bases. The cultivation and management measures of the test materials were basically the same. For each cultivar, 30 fruit of the same size (mature, free of disease and damage) were selected from the east, south, west, and north directions of the plant periphery from three different tree crowns. After being packed in dense bags, the fruit were placed in dry ice and transported to the laboratory without delay. The details of the harvested fruit are shown in Table 2.

Methanol, acetonitrile, acetic acid (HPLC Grade) purchased from Merck company of Germany (Darmstadt, Hesse, Germany). The standard products were purchased from Sigma-Aldrich (St Louis, MO, USA) and BioBioPha (Kunming, Yunnan, China). After being dissolved in dimethyl sulfoxide (DMSO) or methanol, the samples were stored at −20 °C and diluted with 70% methanol before mass spectrometry analysis.

Ultra-performance liquid chromatography–tandem mass spectrometry (UPLC-MS/MS) was performed using a Shim-pack UFLC CBM30A system (Shimadzu, Kyoto, Japan) coupled to a 6500 QTRAP tandem mass spectrometer (Applied Biosystems, Foster City, CA, USA). Other specialized equipment included a ultrapure-water purification system (Millipore, Bedford, MA, USA), an MM 400 mixed ball mill (Retsch, Haan, Germany), a Heto Lyolab 3000 vacuum freeze-drier (Heto-Holten, Allerød, Denmark), an Eppendorf 5427 high-speed freezing centrifuge (Eppendorf, Hamburg, Germany), an electronic balance (MSU224S-100-DU 1/10000, Sartorius, Göttingen, Germany), and an ultrasonic cleaner (KQ-500 DB, Kunshan Ultrasonic Instrument, Kunshan, China).

### 4.2. Treatment and Method

The peel and core of the litchi fruit were removed, and the pulp was vacuum freeze-dried and ground (30 Hz, 1.5 min) to powder in a ball mill. Sample powder (100 mg) was accurately weighed and dissolved in 1.0 mL of 70% methanol for extraction, and the dissolved sample was refrigerated at 4 °C and vortexed three times overnight. After centrifugation (10,000× *g*, 10 min), the supernatant was aspirated, filtered through a 0.22-μm microporous membrane, and stored in an injection bottle for UPLC-MS/MS analysis.

### 4.3. Collection Conditions for Chromatography-Mass Spectrometry

The analytical conditions and related parameters were based on the method established by Chen et al. [40]. The chromatographic conditions were the following: column, Waters ACQUITY UPLC HSS T3 C18 (1.8 µm, 2.1 × 100 mm); mobile phase, ultrapure water (with 0.04% acetic acid), and acetonitrile (with 0.04% acetic acid); gradient elution, water/acetonitrile, 0 min 95:5 *v*/*v*, 11.0 min 5:95 *v*/*v*, 12.0 min 5:95 *v*/*v*, 12.1 min 95:5 *v*/*v*, 15.0 min 95:5 *v*/*v*; flow rate, 0.4 mL/min; column temperature, 40 °C; injection volume, 2 μL. MS analysis was performed with electrospray ionization (ESI) at 500 °C and 5500 V. The curtain gas pressure was 25 psi, and collision-induced ionization was set to high. In the triple quadrupole, each ion pair was scanned according to the optimized declustering potential and collision energy.

### 4.4. Quality Control Samples

Quality control samples (QC) were prepared by mixing the extracts of 5 Different Litchi Cultivars in equal amount, with 3 replicates. The same method was used to treat and detect the samples. In the process of instrument detection, one QC sample was inserted into every 10 samples to investigate the repeatability of the whole analysis process.

### 4.5. Data Analysis

The metabolites were identified based on information in the MWDB V2.0 database and the public database of Metware Biological Science and Technology Company. The primary and secondary mass spectra were analyzed according to MassBank, KNAPSAcK, HMDB, and METLIN. The quantitative analysis of metabolites was carried out by triple-quadrupole mass spectrometry in multiple reaction monitoring (MRM) mode.

After obtaining the MS analysis data of metabolites from different samples, it was processed by using Software Analyst 1.6.3 to integrate the peak areas of all the MS peaks, and the MS peaks of the same metabolite in different samples were integrated and corrected [41]. According to the obtained retention time, mass nucleus ratio and peak intensity, the notes were made, and most of the compounds were further confirmed by using standard materials With FZX as the reference cultivar, a reliable mathematical model was established to analyze the metabolites by using multidimensional statistical analysis. Principal component analysis (PCA), cluster analysis, partial least squares discriminant analysis (PLS-DA), and orthogonal partial least squares discriminant analysis (OPLS-DA) were used to predict the stability and reliability of the model [42,43]. Multi-dimensional statistical analyses (using VIP value), one-dimensional statistical analysis (*p* value), and fold change) were used to predict the stability and reliability of the model. The metabolites with VIP > 1, *p* < 0.05, log2FC ≥ 2, or log2FC ≤ 0.5 were selected as differential metabolites. The different metabolites of each group were clustered by hierarchical clustering. In addition, Graphpad Prism 8.0 and IBM SPSS 25 software were used for statistical analysis. Duncan’s new repolarization difference method was used for comparison between the sample groups, with *p* < 0.05 indicating significant difference.

## 5. Conclusions

In this study, we have performed successfully LC-MS/MS-based metabolic analysis to systematically compare polyphenols differences between five litchi cultivars. It provides comprehensive information on both metabolite compositions and abundances in litchi, a characteristic commercial fruit. A total of 126 polyphenol metabolites were identified, and 15 common differential metabolites and 20 specific differential metabolites to each variety were found. From the results we infer that flavonoids, flavonols, hydroxycinnamoyls, and catechins are the main polyphenol metabolites of litchi pulp; early maturing FZX is a kind of high polyphenol content cultivars, especially in catechins, anthocyanins, flavonols, quinines, and hydroxycinnamoyls. There was a level of correlation between the contents of phenolics and the maturity of litchi cultivars, and the content of phenolics in early maturing litchi cultivars appeared higher than those of mid- to late-maturing cultivars. In view of the close relationship between polyphenols and fruit nutrition, flavor, and resistance, this experiment will provide theoretical basis and reference information for cultivation, breeding, processing, and consumption.

## Figures and Tables

**Table 1:** Statistical table of the number of metabolites of different litchi cultivars in different maturity.

Group Name	All Sig Diff	Down Regulated	Up Regulated
**BTY vs. FZX**	44	37	7
**FC vs. FZX**	39	25	14
**YJQ vs. FZX**	50	44	6
**BYL vs. FZX**	65	49	16

**Table 2:** Sample details of different litchi cultivars.

No	Cultivars	Abbreviation	Maturity Type	Harvest Time	Harvest Site	Longitude and Latitude
**1**	Feizhixiao	FZX	Early-maturing	2 June 2018	Tianhe, Guangzhou	23°9′13″ N, 113°9′13″ E
**2**	Baitangyin	BTY		6 June 2018	Tianhe, Guangzhou	23°9′13″ N, 113°9′13″ E
**3**	Feicui	FC	Medium-maturating	24 June 2018	Gaozhou, Maoming	21°51′25″ N, 110°53′50″ E
**4**	Yujinqiu	YJQ		28 June 2018	Doumen, Zhuhai	22°13′10″ N,113°15′29″ E
**5**	Beiyuanlv	BYL	Late-maturating	5 July 2018	Zengcheng, Guangzhou	23°20′30″ N,113°48′49″ E

## Data Availability

The data presented in this study are available in supplementary material.

## Supplementary Materials

The following are available online. Figure S1: OPLS-DA maps among litchi cultivars. Figure S2: Verification diagrams of OPLS-DA model among litchi cultivars. Table S1: List of 126 polyphenol metabolites. Table S2: Identification information of common difference metabolites. Table S3: Identification information of different metabolites specific to each group of variety.

## Sample Availability

The data used and/or analyzed during the current study are available from the corresponding author on reasonable request.
