# Supplementary material for: Metabolite Differences of Polyphenols in Different Litchi Cultivars (Litchi chinensis Sonn.) Based on Extensive Targeted Metabonomics
Source: Molecules. 2021 Feb 23;26(4):1181. doi: 10.3390/molecules26041181 (PMC7926386; doi:10.3390/molecules26041181)
Supplement: Supplementary file 1 [file molecules-26-01181-s001.pdf]

Supplementary material

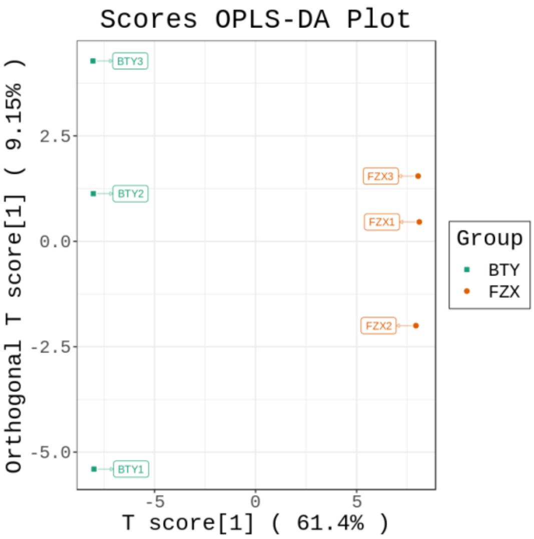

A

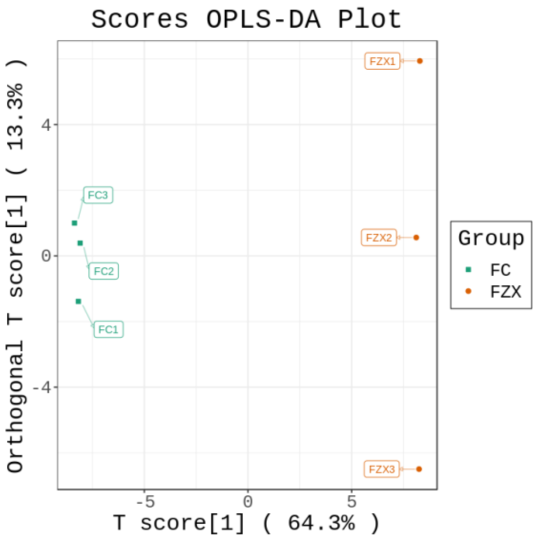

B

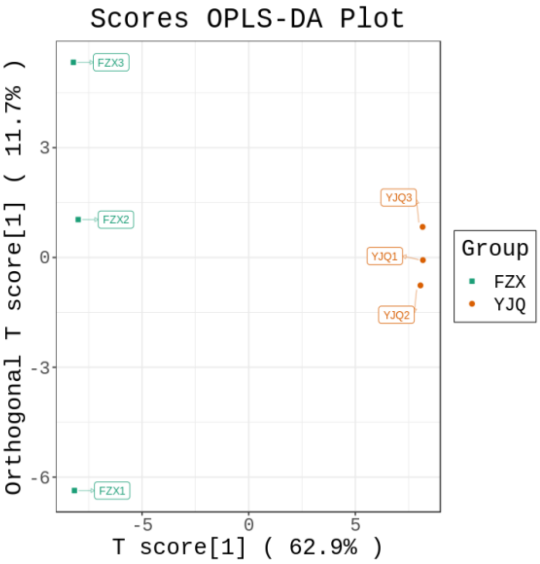

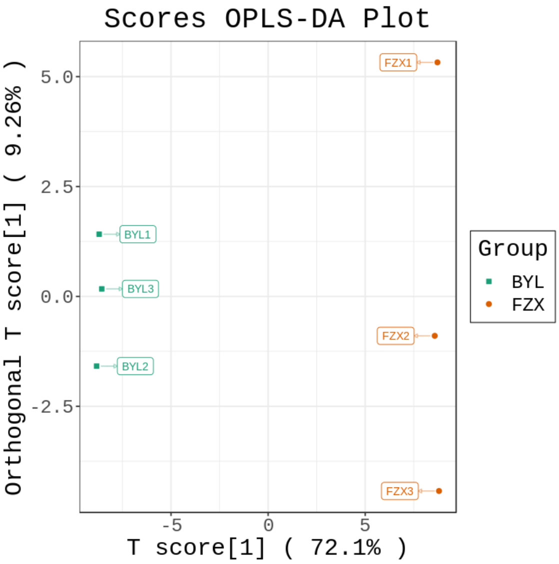

C

D

Figure S1. OPLS-DA diagrams among litchi cultivars

Note: (A) BTY vs. FZX; (B) FC vs. FZX; (C) YJQ vs. FZX; (D) BYL vs. FZX.

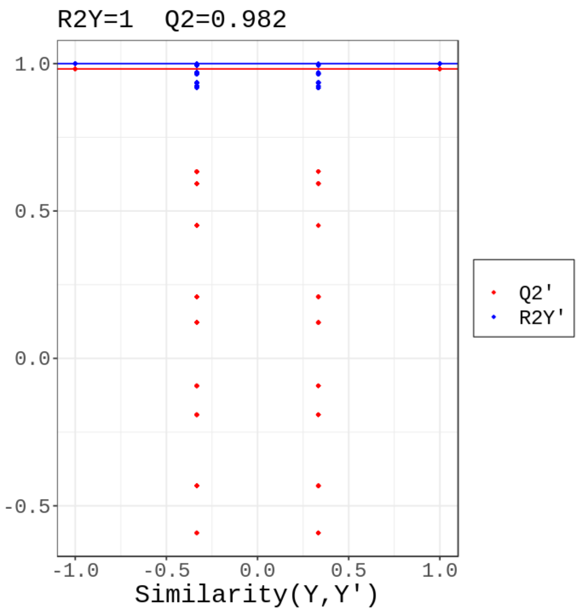

A

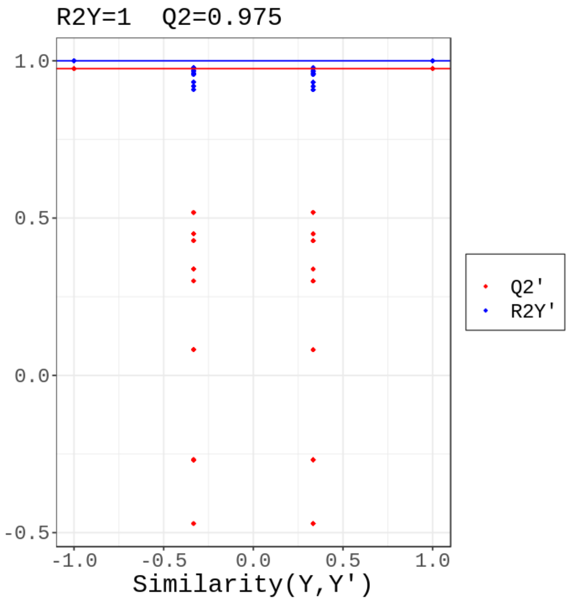

B

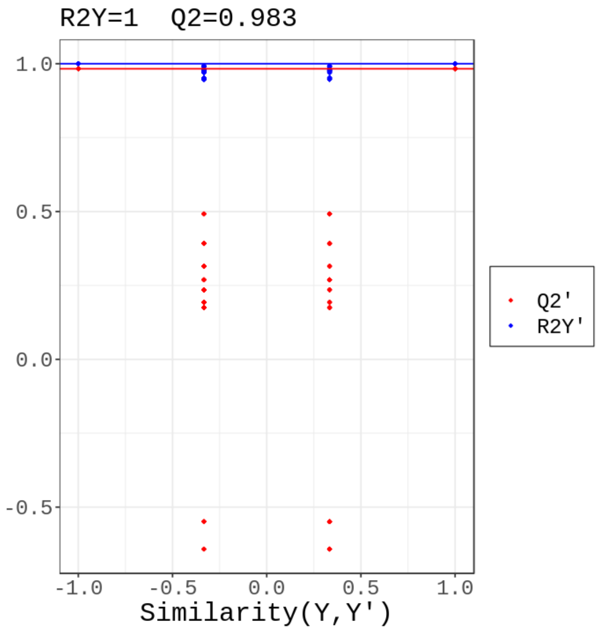

C

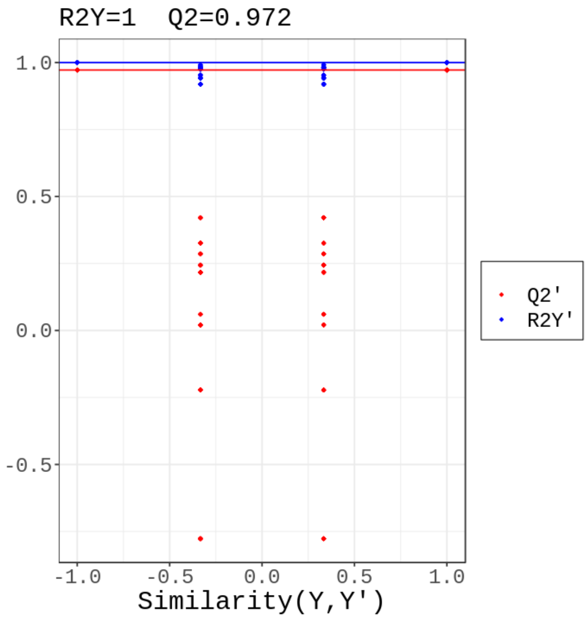

D

Figure S2. Verification diagrams of OPLS-DA model among litchi cultivars

Note: (A) BTY vs. FZX; (B) FC vs. FZX; (C) YJQ vs. FZX; (D) BYL vs. FZX. The horizontal line corresponds to R2Y and Q2 of the original model, the red and blue dots respectively represent R2Y' and Q2' of the model after replacement.

Table S2 Identification information of common difference metabolites

NO.	Compounds	Class	Molecular Weight (Da)	Formula	CAS No.	Rt (min)	VIP	*P*	Tend			
									BTY vs. FZX	FC vs. FZX	YJQ vs. FZX	BYL vs. FZX
1	Tricetin O-malonylhexoside	Flavone	550.00	$C_{24}H_{22}O_{15}$	-	4.52	1.21	0	↑	↓	↓	↓
2	1-O-β-D-Glucopyranosyl sinapate	Hydroxycinnamoyls	386.10	$C_{17}H_{22}O_{10}$	78185-48-5	3.15	1.24	0	↓	↓	↓	↓
3	Luteolin O-hexosyl-O-hexosyl-O-hexoside	Flavone	772.10	$C_{33}H_{40}O_{21}$	-	2.53	1.24	0	↓	↓	↓	↓
4	Ferulic acid	Hydroxycinnamoyls	194.06	$C_{10}H_{10}O_4$	1135-24-6	4.03	1.21	0	↓	↑	↓	↑
5	Quercetin 3-α-L-arabinofuranoside (Avicularin)	Flavonol	434.08	$C_{20}H_{18}O_{11}$	572-30-5	3.96	1.22	0	↓	↓	↓	↓
6	Kaempferol 3-O-rutinoside (Nicotiflorin)	Flavonol	594.16	$C_{27}H_{30}O_{15}$	17650-84-9	3.79	1.24	0	↓	↓	↓	↓
7	Nobiletin	Flavone	402.13	$C_{21}H_{22}O_8$	478-01-3	7.02	1.22	0	↓	↑	↓	↓
8	Tangeretin	Flavone	372.12	$C_{20}H_{20}O_7$	481-53-8	7.5	1.22	0.0096	↓	↑	↓	↓
9	Kaempferol 3-O-glucoside (Astragalin)	Flavonol	448.10	$C_{21}H_{20}O_{11}$	480-10-4	4.03	1.24	0	↓	↓	↓	↓
10	Coniferyl alcohol	Hydroxycinnamoyls	180.08	$C_{10}H_{12}O_3$	458-35-5	3.86	1.14	0	↓	↑	↓	↑
11	Quercetin 4'-O-glucoside (Spiraeoside)	Flavonol	464.10	$C_{21}H_{20}O_{12}$	20229-56-5	3.8	1.20	0	↓	↓	↓	↓
12	Quercetin 3-O-glucoside (Isotrifoliin)	Flavonol	464.10	$C_{21}H_{20}O_{12}$	21637-25-2	3.81	1.21	0	↓	↓	↓	↓
13	Kaempferol 3-O-galactoside (Trifolin)	Flavonol	448.10	$C_{21}H_{20}O_{11}$	23627-87-4	3.99	1.24	0	↓	↓	↓	↓
14	p-Coumaryl alcohol	Hydroxycinnamoyls	150.07	$C_9H_{10}O_2$	3690-05-9	3.5	1.24	0	↓		↓	↑
15	p-Coumaraldehyde	Hydroxycinnamoyls	148.05	$C_9H_8O_2$	2538-87-6	4.12	1.22	0	↓		↓	↓

Table S3 Identification information of different metabolites specific to each group of cultivars

**Group**	**Compounds**	**Class**	**Molecular Weight (Da)**	**Formula**	**CAS No.**	**Rt (min)**	**VIP**	**Log2FC**	**Type**
BTY vs. FZX	Syringetin 7-O-hexoside	Flavone	508.10	$C_{23}H_{24}O_{13}$	-	4.10	1.27	3.42	↑
	Sakuranetin	Flavone	286.08	$C_{16}H_{14}O_5$	2957-21-3	6.92	1.23	-1.51	↓
	Dihydromyricetin	Flavonol	320.05	$C_{15}H_{12}O_8$	27200-12-0	3.47	1.24	1.01	↑
FC vs. FZX	Protocatechuic acid	Catechins	154.03	$C_7H_6O_4$	99-50-3	2.55	1.22	1.75	↑
YJQ vs. FZX	Chrysoeriol 7-O-rutinoside	Flavone	608.10	$C_{28}H_{32}O_{15}$	-	3.99	1.10	1.34	↑
BYL vs. FZX	MethylQuercetin O-hexoside	Flavonol	478.00	$C_{22}H_{22}O_{12}$	-	3.84	1.05	-1.58	↓
	Isorhamnetin O-hexoside	Flavonol	478.20	$C_{22}H_{22}O_{12}$	-	4.11	1.12	-1.71	↓
	Luteolin 3',7-di-O-glucoside	Flavone	610.10	$C_{27}H_{30}O_{16}$	52187-80-1	3.38	1.04	-1.13	↓
	Isorhamnetin 5-O-hexoside	Flavonol	478.20	$C_{22}H_{22}O_{12}$	-	4.00	1.09	-1.49	↓
	Chrysoeriol O-hexosyl-O-hexoside	Flavone	624.10	$C_{28}H_{32}O_{16}$	-	4.12	1.14	-1.60	↓
	Procyanidin A3	Proanthocyanidins	576.10	$C_{30}H_{24}O_{12}$	86631-39-2	2.87	1.15	-1.58	↓
	Tricin 7-O-hexoside	Flavone	492.10	$C_{23}H_{24}O_{12}$	-	4.21	1.14	1.67	↑
	3-O-p-Coumaroyl quinic acid	Quinates	338.10	$C_{16}H_{18}O_8$	1899-30-5	3.46	1.16	1.70	↑
	Apigenin 7-rutinoside (Isorhoifolin)	Flavone	578.16	$C_{27}H_{30}O_{14}$	552-57-8	3.82	1.15	-2.09	↓
	Syringic acid	Hydroxycinnamoyls	198.05	$C_9H_{10}O_5$	530-57-4	3.35	1.12	1.20	↑
	Pinoresinol	Hydroxycinnamoyls	358.14	$C_{20}H_{22}O_6$	487-36-5	5.36	1.16	2.16	↑
	Sinapic acid	Hydroxycinnamoyls	224.07	$C_{11}H_{12}O_5$	530-59-6	3.74	1.03	1.21	↑
	Quinic acid	Quinates	192.06	$C_7H_{12}O_6$	77-95-2	1.13	1.17	-1.54	↓
	Luteolin 7-O-glucoside (Cynaroside)	Flavone	448.10	$C_{21}H_{20}O_{11}$	5373-11-5	3.79	1.06	-1.05	↓
	Tricetin	Flavone	302.04	$C_{15}H_{10}O_7$	520-31-0	4.38	1.13	-1.32	↓
